# Mitochondrial Dysfunction: Cause or Consequence of Vascular Calcification?

**DOI:** 10.3389/fcell.2021.611922

**Published:** 2021-03-16

**Authors:** Kanchan Phadwal, Christina Vrahnas, Ian G. Ganley, Vicky E. MacRae

**Affiliations:** ^1^Functional Genetics and Development Division, The Roslin Institute and The Royal (Dick) School of Veterinary Studies (R(D)SVS), University of Edinburgh, Midlothian, United Kingdom; ^2^Medical Research Council (MRC) Protein Phosphorylation and Ubiquitylation Unit, Sir James Black Centre, University of Dundee, Dundee, United Kingdom

**Keywords:** mitochondria, VSMCs, calcification, mitophagy, oxidative phoshorylation

## Abstract

Mitochondria are crucial bioenergetics powerhouses and biosynthetic hubs within cells, which can generate and sequester toxic reactive oxygen species (ROS) in response to oxidative stress. Oxidative stress-stimulated ROS production results in ATP depletion and the opening of mitochondrial permeability transition pores, leading to mitochondria dysfunction and cellular apoptosis. Mitochondrial loss of function is also a key driver in the acquisition of a senescence-associated secretory phenotype that drives senescent cells into a pro-inflammatory state. Maintaining mitochondrial homeostasis is crucial for retaining the contractile phenotype of the vascular smooth muscle cells (VSMCs), the most prominent cells of the vasculature. Loss of this contractile phenotype is associated with the loss of mitochondrial function and a metabolic shift to glycolysis. Emerging evidence suggests that mitochondrial dysfunction may play a direct role in vascular calcification and the underlying pathologies including (1) impairment of mitochondrial function by mineral dysregulation i.e., calcium and phosphate overload in patients with end-stage renal disease and (2) presence of increased ROS in patients with calcific aortic valve disease, atherosclerosis, type-II diabetes and chronic kidney disease. In this review, we discuss the cause and consequence of mitochondrial dysfunction in vascular calcification and underlying pathologies; the role of autophagy and mitophagy pathways in preventing mitochondrial dysfunction during vascular calcification and finally we discuss mitochondrial ROS, DRP1, and HIF-1 as potential novel markers and therapeutic targets for maintaining mitochondrial homeostasis in vascular calcification.

## Introduction

The process of calcification takes place due to the precipitation of insoluble salts of calcium carbonate and calcium phosphate. In soft tissues, three different types of calcifications are present, dystrophic (occurs in damaged or necrotic tissue), metastatic (hypercalcemia or hyperphosphatemia in blood) and calcinosis (deposition of calcium in the skin, subcutaneous tissue, muscles, and visceral organs) (Black and Kanat, 1985; Booth et al., 2017). Vascular calcification is the most frequent type of soft tissue calcification whereby calcium (Ca) and phosphate (Pi) accumulates in blood vessels and cardiac valves. Vascular calcification is a significant risk factor for cardiovascular morbidity and mortality in patients with end stage renal disease (ESRD), diabetes and atherosclerosis. Currently, there is no pharmaceutical strategy to prevent this pathological process.

Vascular calcification can be classified into three main types based on location: intimal arterial (associated with atherosclerosis), medial arterial (associated with ESRD and diabetes), and aortic valve (associated with calcific aortic valve disease) (Lanzer et al., 2014). The cell types that drive vascular calcification include vascular smooth muscle cells (VSMCs), endothelial cells, calcifying vascular cells, pericytes and valve interstitial cells. These cells types contribute to the formation of calcified nodules and undergo osteogenic phenotype switching. This phenotype switching can lead to either osteoblast or chondrocyte-like differentiation (Hortells et al., 2018). Indeed, both of these cell types are present within small areas of calcification in the innominate arteries in the advanced atherosclerotic lesions of atherosclerosis-prone apolipoprotein E-deficient (*ApoE*^−/−^) mice (Rosenfeld et al., 2000; Davaine et al., 2016). However, advanced atherosclerotic plaques in human patients do not contain osteoblast or chondrocyte-like cells. Instead, they are rich in apoptotic smooth muscle cells, macrophages and cells associated with inflammation (Herisson et al., 2011; Jinnouchi et al., 2020).

Vascular calcification is also associated with apoptosis (Ewence et al., 2008), matrix vesicle release (Chen et al., 2018), and subsequent hydroxyapatite [Ca_10_(PO_4_)_6_OH_2_] deposition within tissues (Lee et al., 2012). The source of hydroxyapatite nucleation has yet to be fully elucidated with three key origins discussed in the literature: (1) Nucleation of hydroxyapatite within the lumen of matrix vesicles and subsequent release into extracellular matrix (Buchet et al., 2013). (2) Hydroxyapatite nucleation within collagen fibrils (Weiner and Traub, 1986; Landis et al., 1996) and (3) Release of mitochondrial-derived vesicles to the extracellular space from Ca-overloaded mitochondria (Boonrungsiman et al., 2012). This process of hydroxyapatite deposition within the collagenous matrix of vascular cells likely shares important biological features with the process of bone formation (Doherty et al., 2003). While majority of the hydroxyapatite originates as calcium phosphate (Bertazzo et al., 2013), a small proportion of it is made up of calcium carbonate, particularly in the human atherosclerotic calcified plaques (Schmid et al., 1980). A group of isoenzymes called carbonic anhydrases facilitates calcium carbonate deposition by reversible conversion of carbon dioxide into bicarbonate (Adeva-Andany et al., 2015). Furthermore, in addition to hydroxyapatite, other sources of arterial calcification have also been reported, like carbonate-rich calcium phosphate dense granules in collagen matrix of calvarial osteoblasts (Nitiputri et al., 2016) and whitlockite, a magnesium-containing crystal [(Ca, Mg)_3_(PO_4_)_2_] observed in a mouse model of uremic arterial calcification (Verberckmoes et al., 2007; Schlieper et al., 2010).

Key molecular determinants of vascular calcification have been identified in health, disease, and pathological ectopic calcification (Giachelli, 1999). These include positive regulators including, msh homeobox-2 like Msx2 (Zhou et al., 2013; Andrade et al., 2017), runt-related transcription factor 2 (Runx2) (Speer et al., 2010), osteogenic morphogen like bone morphogenetic protein-2 (Bmp-2) (Hruska et al., 2005), the phosphate transporter PiT-1 (Li and Giachelli, 2007), and the release of matrix vesicles (Bakhshian Nik et al., 2017). Additionally, local enzymes including tissue-non-specific alkaline phosphatase (TNAP) and phosphate-regulating endopeptidase homolog X-linked (PHEX) are able to degrade calcification inhibitors such as inorganic pyrophosphate (PPi) and osteopontin (Quarles, 2003; Murshed and McKee, 2010; Barros et al., 2013). Failure of this enzyme-substrate relationship can lead to an accumulation or degradation of these key inhibitors of calcification (Reznikov et al., 2020). Furthermore, ankylosis homolog (ANKH) protein and the enzyme ecto-nucleotide pyrophosphatase/phosphodiesterase-1 (ENPP1) inhibit VSMC calcification through enhancing extracellular PPi levels (Ho et al., 2000; Mackenzie et al., 2012; Back et al., 2018; Roberts et al., 2020). The mechanisms through which PPi inhibits vascular calcification include direct binding to hydroxyapatite; increased levels of osteopontin induced via the Erk1/2 and p38 MAPK signaling pathways, and inhibition of TNAP activity (Addison et al., 2007). Moreover, cells expressing ankylosis homolog (ANKH) release PPi and citrate (Ca chelator) along with other tricarboxylic acid cycle (TCA) intermediates including succinate and malate (Hu et al., 2010; Szeri et al., 2019). Interestingly, citrate treatment attenuates vascular calcification (Yao et al., 2018) in a chronic renal failure rat model and reduces high Pi-induced vascular calcification (Ciceri et al., 2016; Ou et al., 2017; Yao et al., 2018). Furthermore, Matrix Gla protein (MGP) binds to Ca and hydroxyapatite to function as a potent inhibitor of vascular calcification (Hauschka et al., 1989; Luo et al., 1997).

Given that vascular calcification is mainly driven by mineral (Ca and Pi) overload and mitochondria are the key regulators of intracellular Ca and Pi levels (Pozzan and Rizzuto, 2000; Pozzan et al., 2000; Rizzuto et al., 2000), their physiology and metabolic function is likely to be effected by this pathological process. It has been demonstrated that an uncontrolled increase in cytoplasmic Ca leads to significant Ca overload within the mitochondria relative to the cytoplasm (Rizzuto et al., 1993). VSMCs containing calcified mitochondria have been reported as early as 1976 (Kim, 1976). Moreover, VSMCs in healthy vessels show calcified mitochondria when exposed to high Ca and Pi concentrations *in vitro* (Shroff et al., 2010). Besides, swollen mitochondria enriched with calcific nodules are a feature of a skeletal muscle injury model of dystrophic calcification in mice (Zhao et al., 2009). Additionally, mitochondrial dysfunction, oxidative stress, altered mitochondrial metabolism and mtDNA damage are reported in common diseases associated with vascular calcification including ESRD (Gamboa et al., 2016; Roshanravan et al., 2016), atherosclerosis (Madamanchi and Runge, 2007) and type 2 diabetes (Nishikawa et al., 2000). Thus, mitochondria offer a novel potential therapeutic target for inhibiting vascular calcification. However, the mechanisms through which Ca and Pi overload impact specific mitochondrial components have yet to be determined.

In this review, we discuss the role of mitochondria in maintaining VSMC function, highlight important mechanisms underpinning the cause and consequence of mitochondrial dysfunction in vascular calcification and consider key mitochondrial markers as targets for developing future therapeutic strategies for the prevention of vascular calcification.

## Role of Mitochondria in VSMC Function

VSMCs within blood vessels require high concentrations of intracellular Ca to maintain their contractile phenotype. This influx of Ca takes place via voltage-activated L-type Ca channels on the plasma membrane. Blocking Ca channels with therapeutics such as verapamil and nifedipine (Ca channel antagonist) reduces calcification both in VSMCs *in vitro* and in patients with coronary calcification (Motro and Shemesh, 2001; Chen et al., 2010). Importantly, mitochondrial oxidative metabolism is the key source of energy for VSMC contraction (Paul, 1983).

VSMCs are the most abundant cells of the tunica media in the vessel wall. Their pivotal role is to maintain vessel structure and function. VSMCs in the vessel wall are often exposed to blood pressure variability, particularly in disease settings e.g., increased blood pressure variability during hypertension (Parati, 2005). Recently, it has been shown that maintenance of physiological blood pressure is required for preserving optimal mitochondrial structure and function in VSMCs (Bartolak-Suki and Suki, 2020). Any fluctuation in blood pressure variability can alter ATP production, increase ROS generation and disturb the mitochondrial network within VSMCs. Mitochondrial fission and fusion proteins including mitofusins (MFN), mitochondrial dynamin like GTPase (OPA1) and dynamin-related protein 1 (DRP1) regulate this mitochondrial reorganization during blood pressure variability (Bartolak-Suki and Suki, 2020).

Additionally, VSMCs maintain a contractile phenotype under physiological conditions, which facilitates blood flow through the arteries. However, under biological stress or injury, the cells differentiate into an osteogenic phenotype with an acquired ability to proliferate, migrate and synthesize extracellular matrix vesicles. These differentiated VSMCs migrate into the intima layer of the vessel wall and can induce arterial stiffening (Lacolley et al., 2017). This acquired ability of VSMCs to proliferate is regulated by mitochondrial outer membrane proteins such as mitofusin-2 (Mfn-2) (Li et al., 2015). Mitofusin-2 is required for mitochondrial fusion (Santel and Fuller, 2001; Chen et al., 2003) and is a primary determinant of oxidative stress-induced VSMC apoptosis (Guo et al., 2007). Increased expression of Mitofusin-2 in VSMCs induces apoptosis through upregulated mitochondrial bcl-2-associated X protein expression, increased Bax/Bcl-2 ratio, cytochrome c release and activation of caspase-9 and caspase-3 (Guo et al., 2007).

Glycoprotein cartilage oligomeric matrix protein (COMP) has a crucial role in maintaining mitochondrial homeostasis in VSMCs. COMP is present both in the extracellular matrix and in the cytoplasm where it binds to mitochondria. Mitochondrial transplantation experiments in VSMCs suggest that mitochondrial COMP regulates the contractile phenotype of VSMCs (Jia et al., 2018). In VSMCS, COMP deficiency leads to decreased mitochondrial membrane potential, defective oxidative phosphorylation and significant mitochondrial fragmentation/fission (Jia et al., 2018). COMP also interacts with prohibitin 2, a mitochondrial inner membrane protein, to maintain mitochondrial homeostasis. Prohibitin 2 plays a key role in maintaining mitochondrial genome stability and mitochondrial respiratory chain complex assembly (Bogenhagen et al., 2003; Strub et al., 2011). Blocking the COMP-prohibitin2 interaction results in a marked reduction in mitochondrial respiration, ATP production and the membrane potential, along with increased VSMC differentiation (Jia et al., 2018). Together these data illustrate the crucial role of mitochondria in the maintenance of VSMC homeostasis for healthy blood vessels.

## Mechanisms of Mitochondrial Dysfunction in Vascular Calcification

**Figure 1:**
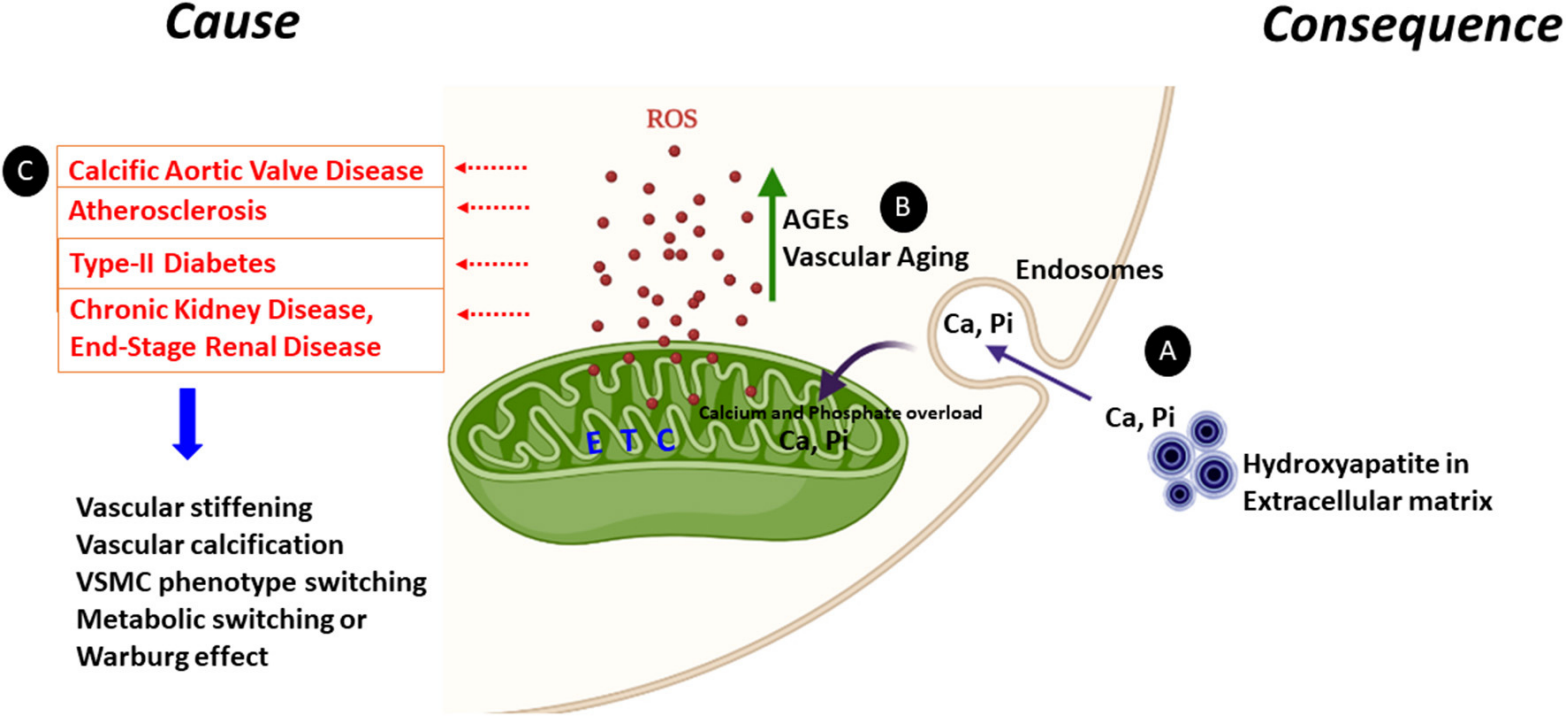
Mitochondrial dysfunction as a cause or consequence of vascular calcification. Increased ROS generation and mineral dysregulation via damaged mitochondrial electron transport chain (ETC) complexes are the likely key cause and consequence of mitochondrial dysfunction observed during vascular calcification. **(A)** Calcium released from the hydroxyapatite crystals from the extracellular matrix can enter the cytoplasm via endosomes and make its way to the mitochondrial matrix causing calcium (Ca) and phosphate (Pi) overload. This calcium and phosphate overload can damage the electron transport chain complexes and generate ROS. **(B)** AGEs are glycotoxins which are elevated in diabetes. AGEs and the receptor for AGEs (RAGE) generate ROS from mitochondria and play a significant role in accelerating the vascular calcification process by enhanced synthesis of extracellular matrix components. Furthermore, ROS production is enhanced in aging vascular cells. **(C)** Enhanced ROS leads to vascular stiffening, vascular calcification, VSMC transition to osteoblast or chondrocyte-like cells and the metabolic switching from oxidative phosphorylation to glycolysis. All these pathways are commonn denominators in pathologies including calcific aortic valve disease, atherosclerosis, type-II diabetes, CKD, and ESRD.

The mechanisms underlying mitochondrial dysfunctional during vascular calcification have yet to be elucidated. Indeed, current literature suggests mitochondrial dysfunction as both a cause and consequence of vascular calcification, as discussed below (Figure 1).

### Mitochondrial Dysfunction as a Cause of Vascular Calcification

Vascular calcification is associated with cardiovascular diseases, metabolic disorders hereditary conditions and renal dysfunction. Vascular calcification is also induced by advanced glycation end products (AGEs) and oxidative stress caused by ROS, which are by-products of aerobic metabolism (Nowotny et al., 2015; Zhu et al., 2018). Excessive ROS production causes oxidative stress, which is heavily implicated in the initiation and progression of vascular calcification, notably during the osteogenic transdifferentiation of VSMCs. Here we discuss the role of mitochondrial dysfunction as a cause of vascular calcification in different calcification pathologies and the key mechanisms potentially driving the calcification process.

#### Calcific Aortic Valve Disease

Calcific aortic valve disease starts with fibrotic thickening followed by extensive calcification of valve leaflets. This leads aortic stenosis and subsequent obstruction of left ventricular outflow. Currently, aortic valve replacement by surgery is the only available treatment for degenerative aortic stenosis.

Recent studies have revealed key evidence for a role of mitochondrial dysfunction in the onset and advancement of calcific aortic valve disease. Mitochondrial (mt) DNA haplogroup analysis in a cohort of aortic stenosis patients has revealed an increased presence of haplogroup H, suggesting haplogroup H as a risk factor in developing aortic stenosis (Serrano-Teruel et al., 2019). Indeed, mitochondrial haplogroup H is an established risk factor for diseases including ischemic cardiomyopathy and idiopathic dilated cardiomyopathy and is associated with increased oxygen consumption and electron transport chain-coupled ROS release (Fernandez-Caggiano et al., 2012, 2013).

Interestingly, increased accumulation of ROS in the form of hydrogen peroxide (H_2_O_2_) is reported in human stenotic aortic valves (Miller et al., 2008) and is implicated in the progression of calcific aortic valve disease (Liberman et al., 2008). Inversely, treating VSMCs with non-toxic concentrations of hydrogen peroxide (H_2_O_2_) induces calcification with a concomitant increase in osteogenic markers including TNAP, collagen type I alpha1 (Col Iα1), Runx2 and osteocalcin (Byon et al., 2008). However, shRNA -mediated silencing of Runx2 abrogates (H_2_O_2_) induced VSMC calcification, suggesting a pivotal role for Runx2 in regulating oxidative stress-mediated vascular calcification (Byon et al., 2008).

Importantly, a metabolic shift from fatty acid to glucose utilization is seen in patients with aortic stenosis. Cardiac biopsy samples taken from these patients during valve replacement surgery show a decrease in expression of proteins involved in fatty acid transport, β-oxidation, the TCA cycle and oxidative phosphorylation, with a concomitant upregulation of glycolysis mediators including GLUT1 and 4 proteins (Heather et al., 2011).

#### Atherosclerosis

Atherosclerosis is a major cause of stroke, myocardial infarction and angina, whereby arteries become clogged with plaques rich in fat, cholesterol and Ca (Ross, 1999; Abedin et al., 2004). Vascular calcification, endothelial dysfunction, intimal thickening and inflammation are the main features of atherosclerosis (Abedin et al., 2004; Spagnoli et al., 2007; Tesauro et al., 2017). Mitochondrial dysfunction in terms of mtDNA damage, increased mitochondrial ROS production and dysfunctional oxidative phosphorylation are present in both human and mouse models of atherosclerosis (Ballinger et al., 2002). Mitochondrial dysfunction-induced apoptosis can lead to plaque rupture thus accelerating atherosclerotic lesion formation (Madamanchi and Runge, 2007).

Recent clinical studies have reported increased mtDNA damage in the peripheral blood mononuclear cells from human atherosclerotic patients (Fetterman et al., 2016). The underpinning role of mtDNA damage in atherosclerosis has been interrogated in polymerase-γ proof reading deficient *ApoE*^−/−^ mice (*polG*^−/−^*/ApoE*^−/−^) (Yu et al., 2013). These mice display defective exonuclease activity of mtDNA polymerase and thus accumulate mtDNA mutations and deletions, leading to mtDNA damage (Trifunovic et al., 2004). This study revealed that mtDNA damage fuels atherosclerosis and generates higher-risk plaques. VSMCs from *polG*^−/−^*/ApoE*^−/−^ mice exhibited reduced ATP, reduced proliferation and increased apoptosis and early senescence. Furthermore, monocytes from *polG*^−/−^*/ApoE*^−/−^ mice showed increased release of interleukin-1β and tumor necrosis factor-α, indicating a pro-inflammatory phenotype. Future studies investigating the vascular calcification phenotype of *polG*^−/−^*/ApoE*^−/−^ mice would be of great interest.

COMP, which has a role in maintaining VSMC mitochondrial homeostasis, prevents VSMC osteochondrogenic transdifferentiation by directly binding to BMP-2 (Du et al., 2011). Calcifying VSMCs and arteries show reduced expression of COMP. Over-expression of COMP ameliorates vascular calcification both *in vivo* and *in vitro* (Du et al., 2011). Conversely, *ApoE*^−/−^ mice lacking COMP show an increase in size of atherosclerotic lesions in the brachiocephalic artery (Bond et al., 2014). Together these studies highlight COMP as an important regulator of vascular calcification and a novel target in the prevention of atherosclerosis.

Extensively calcified aortic tissues and advanced atherosclerotic plaques in patients show an increased expression of mitochondrial carbonic anhydrases (Yuan et al., 2019). Mitochondrial carbonic anhydrases generate ROS via the generation of mitochondrial bicarbonate necessary for oxidative glycolysis. Carbonic anhydrases catalyze the conversion of CO_2_ to HCO_3_^−^ and H^+^ thus increasing cellular acidification (Swietach et al., 2010). This acidification can also precipitate calcium from calcium carbonate, a potential; driver of aortic calcification in CKD patients on calcium carbonate supplements (Phan et al., 2008). Blocking the activity of mitochondrial carbonic anhydrases with acetazolamide alleviates calcification in VSMCs along with the inhibition of inflammatory cytokines associated with atherosclerosis (Yuan et al., 2019). Acetazolamide exerts its effects through the prevention of mitochondrial membrane depolarization and hydrogen peroxide (H_2_O_2_) generation, thus reducing ROS (Yuan et al., 2019).

#### Type-II Diabetes

In type-II diabetic patients, medial calcification is a strong independent predictor of cardiovascular mortality. Compared with non-diabetic subjects, patients with diabetes show increased vascular calcification and upregulation of bone-related proteins, such as osteopontin, type I collagen, bone morphogenetic protein-2 (BMP-2) and TNAP in the medial layer of the vessels (Yahagi et al., 2017). Hyperglycemia, hyperlipidemia and insulin resistance (the hallmarks of diabetes), play an active role in the progression of vascular calcification. Hyperglycemia induces vascular calcification through ROS and superoxide production, either from mitochondria or from alternative sources, such as NADPH oxidase (Nishikawa et al., 2000). Oxidative stress is an important driver of atherosclerotic calcification in diabetes in conjunction with additional factors including endothelial dysfunction, alterations in mineral metabolism, increased inflammatory cytokine production and formation of osteoprogenitor cells. Furthermore, the generation of advanced glycation end products (AGEs) is accelerated in diabetic patients, leading to the subsequent enhancement of vascular calcification through receptor of AGEs (RAGE) (Wei et al., 2013). The role of AGEs in mitochondrial dysfunction is discussed in detail below.

#### Hutchinson-Gilford Progeria Syndrome

Hutchinson-Gilford Progeria Syndrome (HGPS) is a rare premature aging disorder. A recent study investigated the mechanisms underpinning excessive vascular calcification in HGPS patients (Villa-Bellosta et al., 2013). Typically, these patients carry a non-inherited autosomal dominant *de novo* heterozygous mutation at codon 608 of the *LMNA* gene encoding prelamin A. This mutation leads to synthesis of progerin, a truncated version of the lamin A protein. The aorta and aortic valves of these patients showed excessive calcification (Nair et al., 2004; Salamat et al., 2010; Hanumanthappa et al., 2011). Using the *Lmna*^*G*609*G*^ knock-in mouse model, which expresses the human HGPS mutation of progerin, it was shown that *Lmna*^*G*609*G*/+^ VSMCs exhibit reduced mitochondrial ATP production along with reduced function of cytochrome c oxidase (COX), a crucial component of the mitochondrial electron transport chain (Villa-Bellosta et al., 2013). These VSMCs also demonstrate a diminished capacity to synthesize the calcification inhibitor PPi. As ATP is required for the generation of extracellular PPi by ecto-nucleotide pyrophosphatase/phosphodiesterase-1 (ENPP1), reduced ATP availability, due to the dysfunctional electron transport chain may play a direct role in progressing calcification in HGPS, and warrants future investigation.

#### Chronic Kidney Disease

CKD is a gradual loss of kidney function. The reduced kidney function in CKD patients is concomitant with an increase in vascular calcification, which is a significant contributor to cardiovascular mortality in these patients. The mechanisms which regulate calcification in the CKD vessels have yet to be fully elucidated. However, mitochondrial dysfunction is reported within various cells types and tissues during the progression of the disease. Increased oxidative stress is observed throughout the progression of CKD (Dounousi et al., 2006). Additionally, increased skeletal muscle dysfunction is reported in patients with CKD, mediated through decreased activity of mitochondrial TCA enzymes including citrate synthase and hydroxyacyl CoA dehydrogenase (Gamboa et al., 2016). An association between reduced mitochondria number and sarcopenia has also been noted in mouse models of CKD (Tamaki et al., 2014). Furthermore, skeletal muscle from ESRD patients show swollen mitochondria with increased levels of BCL2 and adenovirus E1B 19-kDa-interacting protein 3 (BNIP3), an adaptor protein for damaged mitochondria, suggesting enhanced mitophagy. However, the lysosomes from these patients show increased accumulation of lipofuscin, which may reduce their capacity to degrade damaged mitochondria via mitophagy. In addition, the peripheral blood mononuclear cells from these patients show reduced mtDNA copy number and increased lactate accumulation (Gamboa et al., 2016). Taken together, these data show that mitochondrial dysfunction in CKD is prominent in skeletal muscle and PBMCs; however, this needs further investigation in the blood vessels of CKD patients. Interestingly, there is an *in vivo* evidence demonstrating that mitochondria-derived ROS contributes to microvascular dysfunction in CKD patients (Kirkman et al., 2018). In addition, oxidative stress induced vascular calcification and osteoblastic transition of VSMCs is reported in an adenine diet-based rat model mimicking human ESRD (Shobeiri et al., 2010; Yamada et al., 2012; Neven et al., 2015).

#### Advanced Glycation End Products

Advanced glycation end products (AGEs) are post-translational modifications caused by the non-enzymatic binding of free reducing sugars and reactive carbonyls to proteins or lipids. AGEs are glycotoxins with pathological significance in diabetes, aging and several other chronic conditions (Uchiki et al., 2012; Roca et al., 2014). AGEs accumulate in the extracellular matrix and within the cells of the vessel wall, contributing to the development and progression of cardiovascular disease. Elevated levels of AGEs are typically seen in type-II diabetic patients with coronary heart disease, and in the macrophages and VSMCs from the atherosclerotic lesions (Kilhovd et al., 1999). AGE formation can cause a multitude of receptor-mediated and non-mediated harmful effects to accelerate the atherosclerotic process (Basta et al., 2004; Baumann, 2012). Many of these effects have a role in accelerating the vascular calcification process, including enhanced synthesis of extracellular matrix components leading to vascular stiffening (Sims et al., 1996), increased VSMC proliferation (Zhou et al., 2003) and fibronectin production (Sakata et al., 2000). AGEs and the receptor for AGEs (RAGE) generate ROS from mitochondria in the form of free radicals such as superoxide (${\text{O}}_{2}^{-}$) and hydrogen peroxide (H_2_O_2_) and ROS. Generation of ROS leads to a vicious cycle of further ROS production, dysfunctional mitochondria and more AGEs (Nowotny et al., 2015). Comparably, diabetic RAGE^−/−^ mice are protected against excess generation of both mitochondrial and cytosolic superoxide (Tan et al., 2010).

Recently, it has been shown that advanced glycation products promote both calcification and osteogenic switching of VSMCs by hypoxia-inducible factor-1-alpha, (HIF-1α)/ pyruvate dehydrogenase kinase 4, (PDK4) activation (Zhu et al., 2018). Both HIF-1α/PDK4 contribute toward the metabolic shift to glycolysis (Lambert et al., 2010; Jeong et al., 2012). Furthermore, AGEs downregulate p-AMPKα expression and upregulate p-mTOR expression, thus suppressing autophagy and accelerating the process of vascular calcification. Treatment with the mTOR inhibitor rapamycin reverses this effect and reduces osteoblastic differentiation of VSMCs (Liu et al., 2020). It is interesting to speculate that blocking AGE associated autophagy could have an adverse effect on clearance of dysfunctional mitochondria by mitophagy, which can limit the production of ROS and perhaps progression of vascular calcification.

#### Reactive Oxygen Species

Osteochondrogenic transdifferentiation of VSMCs is a key event during vascular calcification and elevated Pi is the most potent inducer of this transdifferentiation. Extracellular elevated Ca adds a synergistic effect to this phenotypic switching (Cui et al., 2017). In addition to Ca and Pi, there are several other known factors which contribute to this transdifferentiation, including bone morphogenetic factors, elevated glucose and AGEs, dexamethasone, vitamin D3, cytokines, oxidized low density lipoproteins and hypoxia (Tintut et al., 2000; Dorai and Sampath, 2001; Razzaque, 2011; Taylor et al., 2011; Alves et al., 2014; Bessueille et al., 2015; Ceneri et al., 2017; Bao et al., 2020; Lim et al., 2020). More recently, the role of ROS as a mediator of osteochondrogenic transdifferentiation has been recognized (Liberman et al., 2008; Zhao et al., 2011; Byon et al., 2016). Using an organic compound rose bengal to generate free radicals such as singlet oxygen and superoxide anions in rats, Minol et al. demonstrated that ROS induced oxidative stress is a trigger for smooth muscle cell migration, transdifferentiation and vascular calcification in atherosclerotic-like focal vascular degeneration (Minol et al., 2017). Furthermore, hypoxia, which is commonly seen in calcifying tissues, elevates mitochondrial ROS formation and osteogenic transdifferentiation in VSMCs (Chandel et al., 2000). Hypoxic ROS generation is dependent on mitochondrial electron transport chain complexes II and III (Chandel et al., 2000; Paddenberg et al., 2003). Studies have suggested an important role for mitochondrial ROS in the stabilization of HIF1-α, the key regulator of cellular and systemic responses to hypoxia (Sato et al., 2005; Hamanaka and Chandel, 2009). Indeed, this effect of mitochondrial ROS is reversed by N-acetyl cysteine, a ROS scavenger, which downregulates the expression of HIF-1α, RUNX2 and osteocalcin proteins and inhibits extracellular matrix calcification (Escobales et al., 2014).

#### Vascular Aging

Cardiovascular diseases are more prevalent in the aged compared to the younger population, with aging being a major risk factor for atherosclerosis. Furthermore, vascular calcification is a hallmark of age-associated vascular stiffness. Some of the key changes seen in aging cells include decline in the function of mitochondrial ETC function, enhanced oxidative stress and increased senescence (Beckman and Ames, 1998; Feng et al., 2001; Van Deursen, 2014). It is likely that oxidative stress and ROS is central to how aging and vascular calcification connect with each other. Perhaps, HGPS patients with accelerated aging, elevated ROS, early atherosclerosis and vascular calcification represent this connection. Indeed, prelamin A has emerged as a novel biomarker of vascular aging (Ragnauth et al., 2010), whereby accumulation of this molecule induces oxidative damage, mitochondrial dysfunction and premature cell senescence (Sieprath et al., 2015). Prelamin A accumulates not only in the blood vessels of older individuals but also in the blood vessels of young CKD patients on dialysis, where it colocalises with senescent and calcifying VSMCs (Olive et al., 2010; Ragnauth et al., 2010; Liu et al., 2013).

Mitochondrial dysfunction, increased ROS generation, increased mtDNA mutation, altered mitochondria fission and fusion processes and decreased ATP production are mutual to both aging and calcifying cells in the vessel walls (Chistiakov et al., 2014; Sobenin et al., 2015). Moreover, aging vascular cells show reduced expression of antioxidant genes such as mitochondrial superoxide dismutase 2 (Collins et al., 2009). Superoxide dismutase 2 binds to oxidative byproducts of oxidative phosphorylation and plays a crucial role in the defense against ROS (Collins et al., 2009; Van Raamsdonk and Hekimi, 2012). Tempol, a superoxide dismutase mimetic reduces ROS production and mitigates aortic stiffness and fibrosis in a diabetes mellitus mouse model (db/db) (Raaz et al., 2015). It also down-regulates Runx2 expression (Raaz et al., 2015) a key positive regulator of vascular calcification. Moreover, diabetes-accelerated calcification is prevented in rats by the antioxidant apocyanin (Brodeur et al., 2014). Use of antioxidant therapy in attenuating the progression of vascular calcification and vascular aging has been successful in animal models; however, the results are less convincing in humans (Fusco et al., 2007; Conti et al., 2016; Rossman et al., 2018). Interestingly, there is increasing evidence suggesting that ROS is only a part of the multifaceted signaling mechanism which works during aging (Doonan et al., 2008; Cabreiro et al., 2011) and warrants further investigation in understanding the progression of vascular calcification.

### Mitochondrial Dysfunction as a Consequence of Vascular Calcification

Over recent years, several studies have clearly demonstrated that VSMCs undergo calcification in response to changes in extracellular Ca and Pi concentrations. Furthermore, it has been established that elevated Ca is a more potent stimulus to induce calcification than elevated Pi, suggesting Ca as a key mediator of VSMC damage and calcification in pathologies including CKD and ESRD (Deluca and Engstrom, 1961; Kowaltowski et al., 1996; Brookes et al., 2004; Oliveira and Kowaltowski, 2004). Patients with ESRD show high circulating Ca and Pi products and develop extensive vascular calcification. Persistent Ca overload induces mitochondrial permeability transition pore opening (Lemasters et al., 2009), which prompts mitochondrial release of Ca and ROS into the cytosol, which, in turn, compromises mitochondrial function. However, the mechanism underpinning the transportation of Ca and Pi inside the mitochondrial matrix and cytosol of calcifying cells remain poorly defined. Here we discuss the actions of Ca and Pi both individually and synergistically as a cause of mitochondrial dysfunction during vascular calcification.

#### Calcium and Phosphate Overload

A number of clinical studies in ESRD patients have shown an association between elevated serum Ca and Pi levels, increased risk of myocardial infraction and vascular calcification (Blacher et al., 2001; Goodman, 2001; Yamada et al., 2007). Although, the enrichment of Ca crystals within the mitochondrial matrix during the *in vitro* vascular calcification process has been extensively reported (Gonzales and Karnovsky, 1961; Martin and Matthews, 1970; Sutfin et al., 1971; Kristian et al., 2007; Shroff et al., 2010; Boonrungsiman et al., 2012). Albeit, it remains to be investigated whether ESRD patients have elevated cytoplasmic or mitochondrial Ca or Pi levels. Potentially during vascular calcification, diffusion of Ca into the mitochondrial matrix could be a cellular survival mechanism required for prevention of Ca burden in the cytoplasm strewn from the CaPi-enriched extracellular surroundings. However, prolonged mitochondrial Ca overload is the ultimate trigger to initiate apoptosis through MPTP openings, both of which are observed during late stages of vascular calcification (Zhu et al., 2019; Chen et al., 2020).

VSMCs calcified under high Pi conditions *in vitro* show cytosolic release of cytochrome C, increased caspase 9 and caspase 3 activity, and apoptosis (Kim et al., 2012; Chang et al., 2017). *In vitro* experiments from our laboratory have shown that VSMCs cultured in high P_i_ medium show increased expression of glycolytic enzymes including glucose transporter 1 (Glut1), hexokinase 1 (Hex1), and Pdk4 (Rashdan et al., 2020). This increase is associated with reduction of ATP-linked respiration, spare respiratory capacity, and maximal respiration in VSMCs (Rashdan et al., 2020), suggesting that Pi overload can block mitochondrial respiration and force the calcifying VSMCs into glycolysis for energy needs. Mitochondria isolated from rat brain, liver and heart following exposure to high concentrations of Pi, show increased ROS production (Oliveira and Kowaltowski, 2004). These mitochondria display a reduced mitochondrial matrix pH and altered mitochondrial membrane potential (Oliveira and Kowaltowski, 2004). It has been shown that Pi is capable of entering the mitochondrial matrix through a Pi/OH^−^ exchanger and buffering intramitochondrial pH (Kaplan et al., 1986). Increased ROS production is also observed in calcifying aorta of CKD rats fed on a diet rich in Pi, Ca, and vitamin D (Agharazii et al., 2015). These studies suggest that high concentrations of Pi is capable of generating ROS, however, the presence of Ca and Pi together can amplify ROS generation, causing irreversible mitochondrial damage (Kowaltowski et al., 1996). Similarly, Ca and Pi synergistically have a more detrimental effect on mitochondrial function. For example, the opening of MPTPs in the presence of excess Ca becomes an irreversible process in the presence of increased Pi (Kowaltowski et al., 1996). Moreover, a recent study in cardiac mitochondria has revealed that its the CaPi precipitates rather than Ca overload which inhibits complex I activity and oxidative phosphorylation, and the associated reduction in ATP synthesis (Malyala et al., 2019). Together, these studies suggest that elevated levels of Ca and Pi together may play a crucial role in inducing mitochondrial damage during the calcification process. We therefore hypothesize that Ca and Pi overload is a critical trigger for mitochondrial damage and elevated ROS in patients with vascular calcification pathologies.

## Mitochondrial Quality Control in Vascular Calcification

To maintain mitochondrial homeostasis a healthy cell needs to remove damaged mtDNA and the dysfunctional depolarised mitochondria. The cell carries out this function by “mitophagy,” a selective form of autophagy, which degrades these superfluous and damaged mitochondria and its components. However, the role of autophagy and mitophagy in vascular calcification has yet to be fully understood. Detailed reviews on autophagy and mitophagy can be found elsewhere (Montava-Garriga and Ganley, 2020; Nishimura and Tooze, 2020; Yim and Mizushima, 2020). Here we discuss the role of these processes in mitochondrial quality control during vascular calcification.

### Autophagy in Mitochondrial Quality Control

Macroautophagy (autophagy) is the process of removal of unwanted cellular cytoplasmic contents via double membraned autophagosomes. This unwanted cytoplasmic content is passed over to the lysosome for degradation and further recycling. Recent studies have demonstrated the role of autophagy in protection against vascular calcification (Dai et al., 2013; Shanahan, 2013; Peng et al., 2017; Frauscher et al., 2018; Phadwal et al., 2020). Furthermore, the role of autophagy in mitochondrial quality control in VSMCs, specifically in atherosclerotic plaque development, has been recently investigated through VSMC-specific deletion of the essential autophagy gene *Atg7* in the *Atg7*^*F*/*F*^ *Tagln-Cre*^+^ mouse crossed with the *ApoE*^−/−^ mouse model of atherosclerosis (Nahapetyan et al., 2019). For the first time, this mouse model allowed interrogation of the consequences of defective autophagy in the advancement of atherosclerosis. *Atg7* is an E1-like activating enzyme, which facilitates autophagosome formation and expansion. The *Atg7*^*F*/*F*^ *Tagln-Cre*^+^, *ApoE*^−/−^ mouse showed significantly increased atherosclerotic lesions with increased macrophage number and apoptosis. Moreover, *Atg7*-deficient VSMCs from these mice revealed excessive mitochondrial superoxide sensor reactivity. Mitochondria from these transgenic VSMCs also demonstrated low mitochondrial membrane potential. In addition, clusters of fragmented mitochondria were seen present in the close proximity of the nucleus. These mitochondria also showed reduced respiratory reserve capacity indicating dysfunctional mitochondria and impaired clearance (Nahapetyan et al., 2019). Furthermore, these VSMCs show an accumulation of p62/SQSTM1, PINK1 and Parkin, indicating impaired mitophagy, a process of selective degradation of mitochondria via autophagy. This study therefore suggests that defective autophagy processes in VSMCs result in dysfunctional mitochondria and contribute to the progression of atherosclerosis. Similarly, a recent study has demonstrated that autophagy provides protection against Pi-induced mitochondrial dysfunction in kidney proximal tubular cells (PTECs) (Fujimura et al., 2020). Furthermore, proximal tubule-specific autophagy-deficient mice (*Atg5*^*F*/*F*^: kidney androgen-regulated protein mice) fed on a Pi-enriched diet show reduced mitochondrial respiration and increased oxidative stress, with aggregates of fragmented and swollen mitochondria observed in the cytoplasm, suggesting that damaged mitochondria are not cleared in this mouse model (Fujimura et al., 2020).

Autophagy declines with aging, and conversely enhancing autophagy has been shown to improve lifespan in yeast, mice, drosophila and *C elegans* (Hansen et al., 2018). Furthermore, a strong link between energy metabolism, autophagy induction and aging has been established. During cellular aging, energy metabolism controls autophagic activity via acetyl-CoA (Eisenberg et al., 2014; Marino et al., 2014). Acetyl-CoA works as a sentinel metabolite in metabolic regulation. High nucleocytosolic acetyl-CoA amounts are seen during a “nutrient rich” state, which leads to its utilization for lipid synthesis and histone acetylation. However, during “nutrient depletion,” which also induces autophagy, acetyl-CoA moves toward the mitochondria for synthesis of ATP and ketone bodies (Eisenberg et al., 2014; Shi and Tu, 2015). This shuttling of acetyl-CoA between nucleocytosolic and mitochondrial compartments holds the key to adapt under metabolic stress. Knockdown of nucleocytosolic acyl-CoA synthetase 2 (ACS2) leads to a strong induction of autophagy in chronologically aging yeast, however the loss of pathways for synthesis of mitochondrial acetyl-CoA blocks the autophagic flux, suggesting that mitochondrial acetyl-CoA is crucial for survival during aging. High fat diet is a risk factor for vascular calcification. Interestingly, suppression of either acetyl-CoA carboxylase or acyl-CoA synthetase blocks mineralization in vascular cells (Ting et al., 2011). Acetyl-CoA carboxylase plays a key role in fatty acid synthesis via nucleocytosolic acetyl-coenzyme (Pietrocola et al., 2015). Although the link between suppression of nucleocytosolic acetyl-CoA, autophagy induction and vascular calcification remains to be established in vascular cells, it can be speculated that induced autophagy will have a crucial role in suppressing high fat diet-induced vascular calcification and lipotoxicity (Nicoll et al., 2015).

Furthermore, calcifying VSMCs and calcified vessels of patients with atherosclerosis show increased expression of PDK4 and phosphorylated pyruvate dehydrogenase complex (Lee et al., 2015). Phosphate dehydrogenase complex is a mitochondrial enzyme complex that regulates the conversion of pyruvate into acetyl-CoA, whereas PDK4 inhibits this complex by phosphorylating one of its subunits. Under normoxic conditions, pyruvate produced by glycolysis is transported into the mitochondria and converted into acetyl-CoA by pyruvate dehydrogenase complex (Martinez-Reyes and Chandel, 2020). Acetyl-CoA then enters the TCA cycle. However, under hypoxic conditions, inhibition of pyruvate dehydrogenase complex prevents the conversion of pyruvate into acetyl-CoA, leading to decreased TCA cycle activity in the mitochondria and increased conversion of pyruvate into lactate in the cytosol also known as the Warburg effect (Eyassu and Angione, 2017). As discussed above, this metabolic shift is seen in both the calcified vessels of atherosclerotic patients (Lee et al., 2015) and calcified transdifferentiated VSMCs cultured under high Pi conditions (Rashdan et al., 2020). Recently, it has been demonstrated that PDK4 drives the metabolic reprogramming of VSMCs toward a high rate of glycolysis followed by lactic acid generation in the cytosol (Ma et al., 2020). Inhibition of PDK4 abrogates VSMCs calcification by inducing lysosomal function and autophagy (Ma et al., 2020). In conclusion, enhancing autophagy in vascular calcification pathologies may have a multitude of additional effects including inhibiting the Warburg effect, suppressing saturated fatty acid–induced vascular calcification and aiding cellular survival by clearing away the damaged mitochondria via mitophagy.

### Mitophagy in Mitochondrial Quality Control

Under steady state conditions, dysfunctional mitochondria are identified by the upregulation of PINK1, which recruits Parkin, a cytosolic E3 ubiquitin ligase onto the depolarised outer mitochondrial membrane (Aguirre et al., 2017). Further to this, PINK1 and Parkin activate ubiquitin and poly-ubiquitin chains on the depolarised mitochondria, thus triggering their recognition by autophagy adaptors and receptors including p62/SQSTM1 and LC3 (Light chain 3), or the proteasome for degradation (Sarraf et al., 2013). Several outer mitochondrial membrane proteins such as BCL2 interacting protein 3 (BNIP3), FUN14 domain- containing protein 1 (FUNDC1), and NIP3-like protein X (NIX), function as mitophagy receptors by regulating damaged mitochondrial degradation in response to various stimuli. For example, under hypoxia, BNIP3-induced mitophagy is upregulated in VSMCs following lactate-induced calcification (Zhang et al., 2008; Zhu et al., 2019). Furthermore, BNIP3-induced DRP1-mediated mitochondrial fission is required for mitophagy (Lee et al., 2011). This unique process of removing damaged depolarised mitochondria is a crucial safeguard mechanism and an adaptive metabolic response against accumulation of mtDNA damage, ROS and cell death and is perhaps functional during vascular calcification. Depolarised mitochondria (Elmore et al., 2001) and oxidative stress are also known triggers for mitophagy (Frank et al., 2012). Whilst there is no direct evidence to show that mitophagy is upregulated during vascular calcification, it can be speculated that Ca overload during vascular calcification may trigger a mitophagy response.

A recent study in neuronal cells has shown that Ca binding controls mitochondrial motility on microtubules via mitochondrial Rho-GTPase (RHOT1) (Saotome et al., 2008). Under steady state conditions, RHOT1 mediates mitochondrial movement on microtubules promoting mitochondrial fusion. During Ca overload, RHOT1-mediated motility is blocked, which induces mitochondrial fission via Dynamin 1 Like protein (DNM1L) (Saotome et al., 2008). This important research therefore suggests that triggering mitochondrial fission via Ca overload is required for mitophagy.

Interestingly, pharmaceutical attenuation of vascular calcification by melatonin is attributed to reduced mitochondrial fission, increased mitochondrial fusion and mitophagy (Chen et al., 2020). Furthermore, melatonin increases LC3 and Parkin expression both in high fat diet-treated *ApoE*^−/−^ mice and in oxidized low-density lipoprotein-treated macrophage cells, suggesting activation of mitophagy (Ma et al., 2018). Melatonin treatment also inhibits NLRP3 inflammasome activation in oxidized low-density lipoprotein (ox-LDL) treated macrophages via activation of mitophagy (Ma et al., 2018).

Conversely, knock down of PINK1 in human dental pulp stem cells (hDPSCs) results in impaired osteogenesis (Pei et al, 2018). Possibly, mitophagy is underpinning dual roles within VSMCs by (i) contributing to bone formation via exocytosis of Ca and Pi-filled mitochondrial-derived vesicles packed in autolysosomes to the extracellular matrix and (ii) clearing dysfunctional mitochondria within calcified VSMCs. Further studies employing *ApoE*^−/−^ mice deficient in PINK1 would allow investigation of the direct role of mitophagy in the development of vascular calcification and atherosclerosis.

## Mitochondria and Cell Senescence in Vascular Calcification

Cellular senescence along with dysfunctional mitochondria are hallmarks of aging. Senescence of endothelial cells and VSMCs is a common feature of atherosclerotic arteries (Minamino et al., 2002) and ESRD vessels. Vessels from children with ESRD show increased oxidative DNA damage and increased expression of senescence markers p16 and p21. VSMCs isolated from these vessels show accelerated senescence with increased expression of osteogenic markers and calcification, with a concomitant increase in levels of the circulating osteogenic senescence-associated secretory phenotype (SASP) (Sanchis et al., 2019). Furthermore, human VSMCs cultured in the presence of matrix vesicles isolated from senescent endothelial HUVECs show enhanced calcification (Alique et al., 2017). These matrix vesicles were shown to be enriched with Ca and pro-calcification proteins including annexins A2 and A6 and BMP2 when compared to matrix vesicles from young HUVEC cells (Alique et al., 2017). In a similar study, mitochondrial versican-enriched exosomes from hyperglycemia-stimulated vascular endothelial cells were able to induce calcification and senescence in VSMCs cultured under high glucose conditions (Li et al., 2019), further suggesting a link between vascular calcification and senescence.

Inflammation is a common occurrence in atherosclerotic plaque calcification. Inflammatory cytokines including tumor necrosis factor-α (TNFα) and interleukin 1β (IL-1β) are known inducers of VSMC phenotype switching and calcification (Tintut et al., 2000; Ceneri et al., 2017). Interestingly, a 2020 study has revealed that calcified VSMCs from ESRD patients show upregulation of IL-1β, activation of senescence (p21) and elevated osteogenic markers such as bone morphogenetic protein-2 (BMP-2) (Han et al., 2020). Using human VSMCs, the authors further demonstrated that IL-1β-induced senescence is required for osteoblastic transdifferentiation in VSMCs. In addition, senescence-associated secretory phenotype (SASP) along with the DNA damage response dependent VSMC calcification is triggered by prelamin A in VSMCs isolated from human aorta (Liu et al., 2013). Prelamin A (implicated in HGPS) also induces mitochondrial dysfunction via elevation of ROS in human fibroblasts (Sieprath et al., 2015). However, mitochondrial function was not assessed in the VSMCs in these studies.

Together these experiments provide exciting evidence that the process of vascular calcification is able to induce cellular senescence and vice versa. However, it is not yet known if dysfunctional mitochondria are the drivers of VSMC senescence during the calcification process. Dysregulated mitochondrial Ca homeostasis, altered mitochondrial membrane potential, defective oxidative phosphorylation, reduced ATP generation and increased mitochondrial ROS are the key mitochondrial changes seen in senescent cells. Importantly, implementation of the cellular senescence programme specifically requires oxidative phosphorylation, highlighting mitochondria as the key drivers of senescence (Kaplon et al., 2013; Herranz and Gil, 2016). It has been shown that the use of senolytic compounds such as mitocans that target cells with increased mitochondrial potential selectively kills senescent cells by altering their oxidative phosphorylation levels and mitochondrial membrane potential (Hubackova et al., 2019)_._ Furthermore, elamipretide, a mitochondria-targeted peptide, can partly alleviate kidney cellular senescence in porcine atherosclerotic renal artery stenosis by alleviating oxidative stress, and restoring mitochondrial biogenesis and mitophagy (Kim et al., 2019).

Recent elegant studies in fibroblasts have demonstrated that mitochondria underpin pro-aging characteristics of the senescent phenotype (Correia-Melo et al., 2016). Cellular senescence features including up-regulation of senescence-associated secretory phenotype (SASP), p21 and P16, are observed in a novel parkin-mediated mitochondrial clearance model (Narendra et al., 2008). Comparable studies in VSMCs would greatly advance our understanding of the role of mitochondria-derived cellular senescence in vascular calcification.

## Key Mitochondrial Targets in Vascular Calcification

Current literature suggests that ROS is central to mitochondrial dysfunction during the progression of vascular calcification. Several natural dietary, natural non-dietary and synthetic antioxidant compounds, which can scavenge ROS, are being tested for therapeutic efficacy against vascular calcification in clinical trials (Roman-Garcia et al., 2011; Kim et al., 2012; Chang et al., 2017; Chao et al., 2019).

ROS generation is tightly coupled to mitochondrial ultrastructure changes of fusion and fission. Mitochondrial fusion/fission is linked with several physiological indicators of mitochondrial dysfunction, including loss of mitochondrial membrane potential, decreased respiration and oxidative phosphorylation, metabolic shift toward glycolysis, and accelerated mitochondrial ROS. Drp1 is a key regulator of mitochondrial fission (Twig et al., 2008), and is seen enriched in calcified regions of human carotid arteries (Rogers et al., 2017). *In vitro* studies suggest that DRP1 inhibition can attenuate mitochondrial damage both in calcifying VSMCs and in valve interstitial cells, concomitantly reducing vascular calcification (Rogers et al., 2017). Moreover, compounds such as metformin and melatonin abrogate vascular calcification by inhibiting DRP1-induced mitochondrial fission (Rogers et al., 2017; Chen et al., 2020). Metformin also suppresses diabetes-accelerated atherosclerosis via the inhibition of Drp1-mediated mitochondrial fission (Wang et al., 2017). It is interesting to speculate a three-way effect of metformin on mitochondrial function in diabetes-accelerated atherosclerosis, (1) by inhibiting redox shuttle enzyme mitochondrial glycerophosphate dehydrogenase and thus blocking glucose generation by gluconeogenesis, (2) by attenuating mitochondrial ROS generation by inhibiting mitochondrial complex I, and (3) by inhibition of DRP1-mediated mitochondrial fission (Madiraju et al., 2014; Hur and Lee, 2015).

Inhibition of DRP-1 mediated mitochondrial fission also prevents MPTP opening and cell death in HL-1 cells, a cardiac-derived cell line (Ong et al., 2010). Mitochondrial division inhibitor 1 (mdivi-1), a quinazonilone derivative and a DRP1 inhibitor, is able to attenuate oxidative stress-mediated calcification of murine VSMCs by reducing TNAP expression and calcified collagenous matrix production (Rogers et al., 2017). We hypothesize that DRP1 is a key regulator of mitochondrial damage in vascular calcification and underlying pathologies. We further hypothesize that ROS generated during calcifciation process leads to post-translational modifications of DRP1, which may hold the key to its regulation in vascular calcification pathologies. Indeed, several such post-translational modifications of DRP1 has been reported in various cell types. For example, DRP1 acetylation and s-nitrosylation enhances mitochondrial fragmentation in cardiomyocytes and neurons respectively, whereas oxidoreduction of Drp1 protects against mitochondrial fission and ROS generation in endothelial cells (Cho et al., 2009; Kim et al., 2018; Hu et al., 2020).

Hypoxia-inducible factor-1 (HIF-1) is a crucial regulator of vascular calcification. Increase in ROS levels stabilize and activate HIF-1α, which triggers VSMC transdifferentiation and calcification (Patten et al., 2010; Mokas et al., 2016). Osteocalcin, a bone derived hormone promotes phenotype switching in VSMCs and enhances their glucose metabolism and calcification through a HIF-1α-dependent mechanism (Idelevich et al., 2011). Furthermore, an increased nuclear staining for HIF-1α is observed in a CKD rat model (CKD induced by renal mass reduction) fed on a Ca and Pi-enriched diet (Mokas et al., 2016). Increased HIF-1α expression is accompanied by increased expression of HIF-1 regulated genes, such as vascular endothelial growth factor A (VEGFA) and GLUT-1, and reduced expression of VSMC specific markers including actin alpha 2 (ACTA2), Calponin 1 (CNN1), Smooth Muscle Protein 22 (SM22), and myosin heavy chain 11 (MYH11), triggering VSMC transdifferentiation. Furthermore, in calcified VSMCs, elevated Pi levels activate and stabilize HIF-1 independently of oxygen levels by targeting its oxygen-dependent degradation via the ubiquitin proteasome system (Mokas et al., 2016). Exposure of these VSMCs to a mitochondrial targeted antioxidant (SkQ1) and a mitochondrial complex III inhibitor (stigmatellin) blocks this Pi-induced HIF-1 upregulation and calcification (Mokas et al., 2016); suggesting mitochondrial ROS is required for HIF-1 stabilization in Pi-treated VSMCs.

In conclusion, mitochondrial ROS, DRP1, and HIF-1 are highly attractive markers for vascular calcification pathology and offer novel therapeutic targets to improve mitochondrial function during vascular calcification. Furthermore, targeting inhibitors including Fetuin-A, (to reduce Ca Pi precipitation) and, ENPP1, (to reduce Pi overload) could provide an upstream approach to restoring mitochondrial homeostasis in vascular calcification.

## Concluding Remarks

This is a renaissance period for mitochondrial research in vascular calcification. Mitochondrial dysfunction seen in vascular calcification pathologies is strongly associated with mineral dysregulation, ROS generation, phenotypic switching of VSMCs and metabolic shift from oxidative phosphorylation to glycolysis. It would be beneficial to investigate the sequence of mitochondrial dysfunction in a spatiotemporal manner in vascular calcification models.

Whilst Ca and Pi overload during vascular calcification is a likely cause of mitochondrial dysfunction, the mechanisms of Ca and Pi transport to the mitochondrial matrix from the extracellular matrix remain elusive. Moreover, investigating mitochondrial dysfunction during the progression of vascular calcification in mouse models lacking key calcification regulators e.g., *Runx2* (Lin et al., 2015) and *Enpp1* (Li et al., 2013; Huesa et al., 2015) will further define the mechanisms of mitochondrial dysfunction during this process. Additionally, the employment of fluorescent tags fused to mitochondria such as mitoQC (McWilliams et al., 2016), Keima (Sun et al., 2015) or mitotimer (Hernandez et al., 2013) in vascular calcification murine models including mice with *ApoE* ablation or CKD induction will allow the investigation of mitochondrial architecture, biogenesis and turnover via mitophagy during the progression of calcification.

In conclusion, maintaining mitochondrial homeostasis is crucial to protect cells against vascular calcification. Future therapeutic strategies targeted toward molecular regulation of mitochondrial ROS, DRP-1 and HIF-1 could be successful tools in the prevention of vascular calcification pathology and warrant further investigation.

## Author Contributions

All authors listed have made a substantial, direct and intellectual contribution to the work, and approved it for publication.

## Conflict of Interest

The authors declare that the research was conducted in the absence of any commercial or financial relationships that could be construed as a potential conflict of interest.

## Abbreviations

VSMCs: Vascular smooth muscle cells
ROS: Reactive oxygen species
ATP: Adenosine triphosphate
Ca: calcium
Pi: phosphate
CKD: Chronic kidney disease
ESRD: end stage renal disease
TCA: Tricarboxylic acid cycle
TNAP: tissue-non-specific alkaline phosphatase
Runx2: runt-related transcription factor 2
PPi: inorganic pyrophosphate
Drp1: dynamin-related protein 1
HIF-1: hypoxia-inducible factor-1
AGEs: Advanced glycation end products
COMP: Cartilage oligomeric matrix protein.
